# Tunisian medical students’ attitudes and views of psychiatry:

**DOI:** 10.1192/j.eurpsy.2023.2387

**Published:** 2023-07-19

**Authors:** S. Boudriga, M. Lagha, D. Njah, A. Dakhli, I. Ben romdhane, W. Homri, R. Labbene

**Affiliations:** 1 Psychiatry C, Razi Hospital, Tunis, Tunisia

## Abstract

**Introduction:**

According to the World Health Organization (WHO), there is a chronic shortage of psychiatrists on a global scale. In Tunisia, we only have 287 psychiatric specialists, the equivalent of 0,23 per 10 000 habitants in 2017. There is a strong and urgent need to increase the recruitment and retention of doctors in psychiatry, starting from their young years.

**Objectives:**

We aimed to study medical students’ attitudes and views of psychiatry, and their career choices in psychiatry.

**Methods:**

A systematic random sample of medical from two medical schools anonymously completed a questionnaire, distributed via the internet, covering the mental illness: clinicians’ attitudes (MICA) scale, their choice of psychiatry as a career, and the possible associated factors.

**Results:**

A total of 118 medical students participated in the study, with 50% in the second and first years of medical school. The mean age was 21.00 ± 12.2 years. The sex ratio (M/F) was 0.24. We found a personal history of mental health problems in 33.1% of the students.

The mean score of the attitude of health care professionals towards mental illness was 43.61 ± 8.22 out of 96. The results were moderately positive (lowest possible score 25, highest possible score 67) and the female student’s attitude was slightly –but not significantly more positive than male students (male 46.4, female 42.4).

Negative attitudes were reported about the interactions with people with mental health problems, fear of disclosure to colleagues or friends about mental health problems, and confidence in the capabilities of assessing mental health problems in general medicine. No correlation was observed between the immersion clerkship of psychiatry and the MICA scores.

Of the 118 respondents to this question, 35 (29.8%) were most interested in surgical specialties, 35 (29.8%) in medical specialties, and 10 participants (8.5%) were most keen on psychiatry.

**Conclusions:**

Stigma against psychiatry is widespread among medical students. The negative attitudes about mental health and mental diseases may be addressed through educational programs.

**Disclosure of Interest:**

None Declared

